# Circadian Plasticity of Mammalian Inhibitory Interneurons

**DOI:** 10.1155/2017/6373412

**Published:** 2017-03-06

**Authors:** Malgorzata Jasinska, Elzbieta Pyza

**Affiliations:** ^1^Department of Histology, Jagiellonian University Medical College, 7 Kopernika Street, 31-034 Krakow, Poland; ^2^Department of Cell Biology and Imaging, Institute of Zoology, Jagiellonian University, 9 Gronostajowa Street, 30-387 Krakow, Poland

## Abstract

Inhibitory interneurons participate in all neuronal circuits in the mammalian brain, including the circadian clock system, and are indispensable for their effective function. Although the clock neurons have different molecular and electrical properties, their main function is the generation of circadian oscillations. Here we review the circadian plasticity of GABAergic interneurons in several areas of the mammalian brain, suprachiasmatic nucleus, neocortex, hippocampus, olfactory bulb, cerebellum, striatum, and in the retina.

## 1. Introduction

Many aspects of mammalian behavior and physiology show circadian rhythmicity. The circadian rhythms, with a period about a day (*circa*, around and* dies*, day), are generated by the central clock or pacemaker that in mammals is located in suprachiasmatic nuclei (SCN) of the hypothalamus. Under day/night conditions the pacemaker is entrained by light or other cyclic environmental cues (so-called Zeitgebers) and produces oscillations with 24 h period. Circadian information generated by the pacemaker is delivered to other brain regions and to peripheral clocks located in various internal organs. The pacemaker neurons of the SCN exhibit circadian rhythm in electric activity and expression of clock genes which together with their proteins constitute the molecular mechanism of the clock. This mechanism is based on several transcriptional/translational feedback loops, negative and positive [1, 2], and was observed not only in SCN, but also in the majority of mammalian tissues [3]. In mammals the main negative feedback loop begins when two transcription factors CLOCK (CLK) and BMAL1 dimerize and bind to E-box sequences of promoters of their target genes Per1 (Period), Per2, and Per3 and Cry1 (Cryptochrome) and Cry2, driving their rhythmic expression. Next, in the cytoplasm PER and CRY proteins form heterodimers and translocate to the nucleus where they stop transcription of their own genes by inhibiting CLK and BMAL1 transcription factors. This loop is regulated by many posttranscriptional and posttranslational processes and interacts with other loops and control cyclic expression of other, so-called clock-controlled genes (ccg). This mechanism maintains spontaneous oscillations and is able to synchronize precisely to the changeable environment and to control cellular processes by cyclic expression of ccg and other processes [4–6].

Clock neurons in the SCN besides generating circadian oscillations also show various circadian rhythms in physiology and morphology. Similar cellular circadian rhythms have also been observed in many interneurons located in various brain regions.

Interneurons, including those which display circadian rhythmicity, constitute very diverse groups of cells with different physiological, molecular, and morphological characteristics. They allow communication between principal neurons and participate in the formation of all neural circuits. Most of them have short axons and form local circuits but this is not a rule. Hippocamposeptal neurons [7] and interneurons in the medial entorhinal cortex [8, 9] innervate distant targets and are called long-range interneurons. In addition to the main neurotransmitters: glutamate, gamma-aminobutyric acid (GABA), or acetylcholine (Ach) interneurons also release a wide range of neuropeptides such as vasoactive intestinal polypeptide (VIP), neuropeptide Y (NPY), cholecystokinin (CCK), somatostatin (SOM), and many more [10, 11].

In vertebrates, the majority of interneurons are inhibitory and they mainly release GABA. However, GABA can also act as an excitatory neurotransmitter. During brain development, GABA is the main excitatory neurotransmitter acting by GABA A receptors and cooperating with glutamate [12–16]. The effect of GABA includes both excitatory and inhibitory responses which depend on the neuronal network state [17].

Interestingly, GABAergic excitation mediated by GABA A receptors was also observed in the mature brain: in hippocampal interneurons and pyramidal cells [18, 19] as well as in SCN [20, 21].

In the hippocampus, different changes in cell membrane potential—hyperpolarization or depolarization—during GABA neurotransmission were due to different amounts of the released neurotransmitter, which were associated with diverse ions mediating GABA effect: chloride and bicarbonate anions in case of low and high concentration of GABA, respectively [22, 23]. Moreover, the excitatory response to GABA in the pyramidal cells was caused by a transient increase of extracellular K^+^ driven by the K^+^-Cl^−^ cotransporter isoform 2 and was preceded by intracellular increase of Cl^−^ [18, 19, 24].

GABA can also act as an excitatory neurotransmitter in the SCN interneurons [20, 21, 25–27]. According to Wagner et al. [20] GABA increases the firing frequency of neurons during the day and decreases it at night. In light/dark conditions, the transmission is mediated by GABA A receptors linked with chloride channels. It has been postulated that the observed dual effects of GABA result from diurnal oscillations in intracellular chloride concentration in the postsynaptic neurons that is high during the day and low at night. Gribkoff at al. [28] using the similar concentration of extracellular potassium ions repeated the previous study of Wagner et al. [20] and observed only the inhibitory effect of GABA during the subjective day and the subjective night. Hee et al. [25] have proposed that GABA-evoked excitatory response is dependent on Na(+)-K(+)-2Cl(−) cotransporter isoform 1, which is thought to be responsible for the switch between depolarization and hyperpolarization evoked by GABA [14]. In contrast to the results of Wagner et al. [20], they reported the increase of GABA-mediated excitation during the night that was also observed in other studies [21, 27]. This discrepancy probably results from different recording technique (whole patch clamp versus perforated patch clamp) and it seems that the excitatory action of GABA in the SCN occurs throughout day and night but increases during the night.

Some studies showed that the shift from inhibitory to excitatory effects of GABAergic transmission is characteristic predominantly for the dorsal part of the SCN, while neurons in the ventral part show purely inhibitory response [29, 30]. However, other results indicated a possibility of the excitatory action of GABAergic transmission in both parts of the SCN [27]. The conflicting results are probably due to different conditions of the experiments: time of the day, activity state of neurons, or recording techniques.

In the regions of the brain which exhibit diurnal and circadian rhythms, that is, in the SCN, retina, cortex, hippocampus, olfactory bulb, cerebellum, and striatum, inhibitory interneurons occur in different proportions. Even if they are in the minority, modulation or deficits of their activity can have dramatic consequence for network function. GABAergic interneurons not only are responsible for the maintenance of excitatory/inhibitory balance in the local circuits [31–33] but also may generate intrinsic oscillations to synchronize the activity of other neurons [34–38].

In SCN, GABA seems to synchronize [35, 38] and desynchronize the neuronal networks [39]. The final GABA action, synchronization or desynchronization, seems to depend on the response to GABAergic transmission, excitatory or inhibitory, respectively, as well as to the previous excitation status of the target neurons [29, 40].

The desynchronization of neuronal networks in the SCN and in the clock-regulated regions outside the SCN, followed by loss of circadian rhythmicity, is characteristic for age-related circadian dysfunctions [41–43]. They probably result from the disruption of VIPergic and GABAergic signaling in these regions [44–46].

Changes of circadian plasticity, which are correlated with disturbances in cyclic GABA production/release by interneurons (mainly cortical), are associated with some neurological/psychiatric disorders. These disorders display the loss of circadian rhythms and dysfunction of excitatory/inhibitory balance affecting cognitive performance [47] and include Alzheimer's disease [48, 49], Parkinson's disease [50], and schizophrenia [51, 52], as well as autism and epilepsy [53]. Moreover, extrinsic changes in the circadian rhythm such as jet lag, social jet lag, or rotating shift work seem to be correlated with civilization diseases like obesity [54], heart attacks, and diabetes [55].

The investigations of circadian plasticity related to GABAergic interneurons have been focused on expression/concentration of GABA and on colocalized neuropeptides and GABA receptors, as well as on the number of GABAergic synapses and electrophysiological changes of neurons.

## 2. Suprachiasmatic Nucleus (SCN)

Mammalian SCN located in the anterior part of hypothalamus has been shown to play a role of an oscillator responsible for controlling circadian rhythms in other parts of the brain. SCN is divided into two different regions: dorsal and ventral, containing distinct subpopulations of interneurons. Almost all interneurons in both regions of the SCN are GABAergic [56–58] and provide transmission using both classes of GABA receptors, GABA A and GABA B [59, 60]. The SCN interneurons usually also contain one or more neuropeptides which are colocalized with GABA [61, 62]. The neurons of the ventral region produce VIP (the most common neuropeptide in the SCN), peptide histidine isoleucine (PHI), and gastrin-releasing peptide (GRP) [63]. The dorsal region contains neurons producing arginine-vasopressin peptide (AVP). Via the retinohypothalamic tract, the ventral region receives inputs from photosensitive retinal ganglion cells of which axons make synaptic contacts with both VIP and non-VIP interneurons. The secondary photic pathway, the geniculohypothalamic tract which begins in thalamic intergeniculate leaflet (IGL), also leads to the ventral region of SCN. In addition projections from the midbrain raphe terminate onto VIP and AVP neurons in both SCN regions, although they form more synaptic contacts in the ventral region [64–66].

### 2.1. Electrophysiological Rhythms

The SCN neurons generate self-sustained rhythmic activity with approximately 24 h period and their cyclic neuronal firing is driven by rhythmic gene expression [67–69]. It has also been demonstrated that various parameters of synaptic transmission as well as sensitivity of synapses to neuropeptides or electrophysiological stimulation exhibit circadian or diurnal fluctuations.

The diurnal oscillations of the interneuron conductance were measured in rat SCN slices and the conductance recorded at the beginning of the subjective day was 40% higher than at the beginning of the subjective night [70]. Electrophysiological analyses also showed diurnal rhythmicity in the holding current (required to hold a neuron at the constant voltage of −60 mV) with a peak in the middle of the subjective day and minimum in the middle of the subjective night. These methods allow the direct monitoring of ionic conductances and the observed differences provide evidence that at least two different ion channels (for Na+ and K+) must be engaged in driving the diurnal rhythm.

In turn changes in the degree of synaptic depression were measured by paired-pulse depression (PPD) of synaptic contacts between the SCN interneurons. Short-term synaptic plasticity evoked in this way displayed a clear diurnal rhythmicity [71]. Moreover, there were two types of PPD classified according to differences in response to the stimulus, with shorter or longer intervals and preferential time of PPD occurrence. The type 1 PPD showed stronger inhibition at short interstimulus intervals and appeared at the beginning of the day, while the type 2 was characterized by the strongest inhibition at longer interstimulus intervals and occurred preferentially in the middle of the day. It seems that these two PDD types have also different mechanisms: the first involves depletion of synaptic vesicles and the second one is Ca(2+)-dependent [71].

GABA participates in the regulation of amplitude [72, 73] and phase [74–76] of the SCN rhythms and contributes to the maintenance and coordination of the neuronal rhythmicity [29, 40, 72, 77]. It seems that VIP can influence changes in the degree of synchronization between neurons by modifying the frequency of GABA release [78]. Recordings of spontaneous GABAergic inhibitory postsynaptic currents (IPSCs) showed that GABAergic inhibition was dependent on the SCN region and time of the day. The highest frequency of IPSCs was found in the dorsal SCN at the end of the day and at the beginning of the night under light/dark (LD) conditions [79]. The effect of VIP on GABAergic inhibition, mediated by VPAC2 receptors, could be observed in the whole SCN and was more profound during the day when compared to the night. The IPSCs rhythmicity of GABAergic interneurons also occurred in mice kept in constant darkness (DD), indicating the circadian origin of VIP contribution to GABA transmission [78, 79].

### 2.2. Molecular Rhythms

Circadian rhythmicity of GABA content and GAD activity were detected in the rat SCN under LD and DD conditions, suggesting an endogenous nature of the fluctuations [80]. In LD conditions, GABA concentration increased at night and peaked in the middle of the night, while in DD conditions there was a shift of GABA content pattern and its highest level was observed at the beginning of the night. In contrast, GAD activity pattern was similar in both LD and DD. In addition, content/activity of both GABA and GAD was higher under LD comparing with DD [80], what suggests an influence of light on their levels. However, other studies showed daily [81], but not circadian, rhythm of GAD65 mRNA level in the rat SCN [82] implying that light is necessary for GAD rhythmicity.

In rat SCN, VIP and VIP receptor VPCA2 mRNA levels also showed rhythmicity under LD conditions, with a maximum at night and minimum during the day [83]. A similar pattern of pre-pro-VIP/PHI mRNA level changes was reported by Albers et al. [84]. However, VIP protein concentration was constant during the day/night cycle, although constant light decreased the VIP level [85]. In contrast, Francl et al. [86] observed the maximum of VIP release in the midday under LD 14:10 in the hamster SCN and no VIP rhythmicity in DD conditions. This discrepancy of results concerning VIP release is most likely caused by different methods, light/dark conditions, and/or animal models used in the experiments.

The calbindin- (CalB-) expressing neurons of the hamster SCN exhibited the circadian rhythm of CalB level under both LD and DD conditions, with significantly higher expression during the night and the subjective night [87].

### 2.3. Structural Rhythms

The analysis of synaptic contacts between CalB-immunoreactive axons and AVP cells in the hamster SCN showed an increase in the number of synapses during the night when compared with the day. However, there were no changes in the number of synapses formed by CalB-expressing interneurons on other neurons in the SCN like VIP, CCK, and GRP cells under LD and DD conditions [87].

Postsynaptic densities in the rat SCN synapses formed by optic nerve terminals on interneurons showed changes dependent on light conditions and associated with changes in the number of synapses [88]. Further quantitative analysis of synapses between glutamatergic and nonglutamatergic axons representing the retinal input and VIP neurons revealed an increase in the number of both types of synapses during the day and a decrease at night. In contrast, the density of synapses on AVP neurons was stable during the day and night [89, 90]. Moreover, the area of glial coverage around VIP dendrites also exhibited circadian rhythmicity. It increased at night, probably at the expense of axon terminals on soma and dendrites of VIP neurons, because axon area decreased at night [89]. The circadian changes of glial coverage of AVP dendrites showed an opposite pattern. It decreased during the night and was accompanied by increase in the area of AVP perikarya but not by changes in the number of synapses [89]. Electron microscopic studies have not shown, however, any differences in the morphology of glutamatergic axon terminals between the day and night [90].

Results of studies provide a strong evidence that GABAergic interneurons affected by VIPergic signaling play a significant role in the regulation and synchronization of rhythmicity in the SCN.

## 3. Retina

Inhibitory interneurons of the mammalian retina include amacrine cells and several types of horizontal cells [91]. Amacrine neurons may use both inhibitory neurotransmitters, GABA and glycine, while inhibitory horizontal cells are typically GABAergic [92, 93]. There are also a few interplexiform cells expressing GABA [94]. Amacrine cells are medium to large size neurons that terminate on bipolar cells, ganglion cells, or other amacrine cells using GABA A and GABA C receptors for transmission [95]. In some amacrine cells GABA colocalizes with other neurotransmitters, serotonin, acetylcholine [96], and dopamine [97] or neuropeptides such as substance P [98], parvalbumin (PV), SOM, VIP, and NPY [99, 100]. Similarly, glycinergic amacrine cells can also express CCK [99]. Retinal neurons contain different calcium-binding proteins, distributed according to cell classes and sometimes species-specific. For instance AII types of amacrine cells in the cat retina expresses calretinin (CalR) [101], while in the rat retina it expresses PV [100].

Diurnal changes in GABA concentration and turnover were shown under LD 14:10 in the hamster retina, with a clear minimum in the middle of the day. GAD activity was also lower in the middle of the day. Both rhythms were maintained under DD, indicating circadian origin of GABAergic inhibition [102].

PV expression was studied in amacrine cells of the rat retina under LD conditions. The level of PV exhibited cyclic changes with a minimum during the second half of the day and maximum in the middle of the night. This rhythmicity was retained under DD; however, after three days of DD the amplitude of PV level was reduced [103]. The expression of protein kinase C involved in the control of GABA release in amacrine cells showed the opposite pattern of rhythmicity with a peak in the middle of the day and trough in the middle of the night [104].

These observations suggest that the plasticity of retinal interneurons not only results from the direct influence of light but also is controlled by the circadian clock.

## 4. Neocortex

Inhibitory interneurons constitute about 20% of neocortical neurons and show a wide range of diversity in electrical and molecular properties as well as in morphological structure. They have aspiny dendrites, receive soma-targeted excitatory, and inhibitory inputs and form local connections within cortical columns, although some of them project laterally between adjacent columns [10, 105–107]. Neocortical inhibitory interneurons innervate different subdomains of pyramidal cells as well as other interneurons [10, 108].

Diurnal rhythms of GABA turnover and GABA A receptor activity were found in the whole cerebral cortex of hamster in LD 14:10 with peaks at night [109, 110]. However, there were no differences in GABA turnover during the day/night cycle in hamsters kept under the shorter photoperiod of LD 10:14 [109]. No circadian differences in GABA concentration were detected in visual and sensorimotor regions of the human brain [111]. There were no circadian changes, either, in intracortical excitability of GABAergic interneurons located in the human primary motor cortex [112].

The circadian rhythm in the number of synapses localized on dendritic spines was demonstrated in layer 4 of the mouse somatosensory cortex (barrel cortex) which receives sensory input correlated with the circadian rhythm of locomotor activity of animals [113, 114]. Under LD conditions, the number of inhibitory synapses increases during active (nocturnal) phase of the day/night cycle and achieves a distinct peak in the middle of the night. The increase in the number of inhibitory synapses was even more pronounced under DD conditions [114]. These synapses were formed on dendritic spines which previously, in the light phase of the cycle, had excitatory synapses, which means that circadian plasticity of dendritic spines includes transformation of single-synapse spines into double-synapse spines. Although it is known that the inhibitory terminals in the neocortex are intercortical [115] and there are some candidates for input neurons, for example, double bouquet cells or Martinotti cells [10, 116], the source of input to inhibitory synapses in the barrel cortex is unknown.

As it appears from these studies, somatosensory cortex seems to be the only neocortical region that exhibits circadian plasticity of inhibitory interneurons. Future studies of other neocortical areas are clearly required to extend our knowledge concerning the circadian rhythms in the neocortex.

## 5. Hippocampus

Hippocampal GABAergic interneurons are mainly basket and chandelier cells [117, 118]. They innervate dendrites and cell bodies of pyramidal cells and participate in feedback or feedforward circuits [119]. Apart from interneurons cooperating with principal cells, hippocampus contains the so-called interneuron-selective cells (IS), innervating other interneurons and divided according to their connectivity and neurochemical profile into three types [23]. IS-1 interneurons express CalR and they are preferentially located in CA1 field, where they form clusters of 10–15 cells connected dendrodendritically to each other. They give inputs to CalR, CalB, and VIP neurons [23]. IS-2 interneurons express VIP and terminate onto CalB and other VIP interneurons; moreover, they also can innervate interneurons directly inhibiting pyramidal cells [23, 120]. IS-3 interneurons also include VIP neurons that are connected mainly with CalB and SOM neurons [23, 120]. The interneuron-selective cells are believed to control disinhibition in hippocampal networks [23].

Subnetworks of GABAergic interneurons in the hippocampus may influence excitability of glutamatergic pyramidal cells [23]; they are also engaged in the generation of synchronized network oscillation of hippocampal neurons [121]. Hence, circadian changes observed in CA1 pyramidal cells are probably involved in interneuron activity. During long-term potentiation (LTP) evoked on hippocampal slides of rats and hamsters under LD conditions, the population spike (PS) amplitudes doubled during the day comparing with values at night [122, 123]. However, another study of CA1 region of rat hippocampus showed an opposite effect: enhancement of PS during the night in comparison with the day [124]. A similar observation, that is, greater PS-LTP during the subjective night, was reported in a study of the mouse hippocampus in which the pattern of rhythmicity was retained in DD conditions [125]. Diurnal variations in the decay of LTP, measured as excitatory postsynaptic potentials (EPSP), were recorded in CA1 region of the hippocampus in mice [125] but not in rats [124]. It seems that the above-mentioned inconsistency of PS-LTP and EPSP-LTP results might have been caused by different protocols of LTP evocation. The application of GABA A receptor antagonist (gabazine) enhanced PS-LTP during the day to the level observed at night.

These results suggest that the main inhibitory transmission of hippocampal interneurons during LTP is mediated by GABA A receptors and is controlled by the circadian clock [124].

## 6. Olfactory Bulb

Two main subpopulations of GABAergic interneurons in olfactory bulb include periglomerular and granular cells [126, 127]. Granular cells have no axons and they release GABA using specific long dendritic spines forming dendrodendritic reciprocal synapses with mitral cells [126, 128]. Periglomerular cells terminate onto mitral and tufted cells [126] and are more effective in inhibition of mitral cells than granular cells. They are probably responsible for baseline inhibition, while granular cells are involved in facilitating this inhibition [129]. A fraction of periglomerular cells coexpress GABA and dopamine [130].

VIPergic interneurons were observed in the external plexiform layer, periglomerular layer, and granule cell layer of mouse olfactory bulb, while expression of VIP receptor (VPAC2) was detected in mitral and external tufted cells [131].

Intrinsic circadian rhythms of firing rate and clock gene expression were observed in olfactory bulb in vitro [132]. In mice, the circadian rhythm of odor perception sensitivity was detected in the absence of environmental time cues, and it was depended on clock gene expression [133]. Moreover, the rhythm persisted when circadian rhythms were not present in the SCN [134] or when the SCN was ablated [135]. Hence, olfaction seems to be regulated by a circadian clock that is independent of SCN. The circadian rhythmicity in odor perception sensitivity accompanied by the cyclic clock gene expression shows maximum in the early night [133].

A recent study of mouse olfactory bulb demonstrated rhythmic VIP release by interneurons of the external plexiform and periglomerular layers, with the maximum in the middle of the night. Hence, VIPergic interneurons in the olfactory bulb seem to be well positioned to provide circadian coordination of cyclic processes and it is highly possible that they play a similar role to that of VIPergic interneurons in the SCN [131].

Studies on GABAergic transmission and its relation to circadian rhythms in the olfactory bulb could probably complete a picture of the postulated “olfactory clock.”

## 7. Cerebellum

Cerebellar cortex contains several types of inhibitory interneurons mainly releasing GABA except for Golgi cells that also coexpress glycine. Golgi cells provide feed backward inhibition to excitatory interneurons, granule cells [136]. Basket and stellate cells are situated in the molecular layer of cerebellum and cooperate with Lugaro cells, another type of inhibitory interneurons, in the inhibition of Purkinje cells [137, 138]. Inhibitory interneurons in the cerebellum contain different calcium-binding proteins which can be species-specific [139, 140].

Cerebellum contains a circadian food entrainable oscillator that is responsible for the rhythm in food anticipatory activity and is synchronized by the mealtime during scheduled feeding in mammals [141]. It does not seem to be influenced by the light/dark cycle, although the time of clock gene expression in the cerebellum suggests a relationship between the SCN and the cerebellar oscillator [142].

Involvement of inhibitory interneurons in cerebellar oscillator system is unclear. Diurnal changes of GABA turnover rate were demonstrated in hamster cerebellum in LD 14:10 and LD 10:14. Under both light/dark conditions, the maximum of GABA turnover rate was achieved at night [109]. Taking into account that Purkinje cells, the principal cerebellar neurons, are also GABAergic, the actual contribution of interneurons to the cyclic changes in GABA is uncertain. However, the key role of inhibitory interneurons in all brain structures related to the circadian clock might suggest that also in the cerebellum the observed rhythm of GABA turnover is evoked by rhythmic action of interneurons.

## 8. Striatum

GABAergic interneurons in striatum constitute at least 3-4% of neurons [143, 144]. They are medium-sized aspiny neurons with varicose dendrites [145] and are divided into different classes. The main class of striatal interneurons are fast-spiking (FS) cells expressing PV [143, 144] that are involved in the synchronization of striatal inhibition [146]. The second class includes interneurons containing NPY, SOM, and nitric oxide synthase colocalized with CalB. They play a role in the control of local flow of blood and in the modulation of principal cell activity in a feedforward fashion [144, 146, 147]. The third class of GABAergic interneurons are CalR-immunoreactive cells [143, 148]. A distinct class of striatal interneurons are cells which corelease GABA and dopamine [149].

Although striatal interneurons are not numerous, there is strong evidence that they play an important role in the regulation of intrastriatal inhibition.

Circadian GABA fluctuations were detected in the rat striatum under LD conditions with a peak at the end of the day. This rhythmic pattern did not change in DD and LL (constant light) conditions, suggesting that GABA rhythmicity in this region is light-independent [150].

The influence of GABAergic transmission on the dopaminergic system and vice versa is particularly interesting, because its dysfunction in the striatum seems to be critical for Parkinson's disease and a wide range of mood disorders. It is also associated with drug and alcohol addictions [151]. All these disorders are characterized by disruptions of the circadian rhythmicity [151–153].

All stages of Parkinson's disease (PD), according to Braak's model [154], display disturbances in GABAergic transmission in the striatum, especially in Ca^2+^/GABA mechanism that plays the role in stabilizing neuronal activity [155]. The changes in Ca^2+^/GABA ratio lead to the weakening of blood-brain barrier and the intracellular accumulation of Ca^2+^ [156]. The GABAergic system protects neurons from toxicity by the accumulation of intracellular Ca^2+^ via both GABAergic receptors and glial networks [155]. Patients with PD have changes in circadian rhythms including disruptions of the sleep-wakefulness rhythm [157] as well as of rhythmic changes in heart rate [158] and blood pressure [159]. PD-associated changes in the clock gene expression were also reported [160]. However, it is uncertain what appears first—the circadian rhythm disruptions or PD symptoms [50].

The reward system directly involved in drug and alcohol addictions includes the ventral striatum, especially neurons in the core and shell of the nucleus accumbens [161, 162]. The ventral striatum seems to be mainly engaged in reward prediction [163] and is also activated during reward anticipation [164]. This striatal region is also associated with addictive behaviors such as context-induced reinstatement of cocaine [165] and alcohol seeking [166], while the dorsolateral striatum is linked with reinstatement of methamphetamine seeking [167]. During conditioned methamphetamine pairing, a decrease in the level of GABA A receptor *α*1 subunit was observed in the dorsal striatum, suggesting an involvement of GABA A receptors in methamphetamine-associated rewarding memory mechanism [168]. On the other hand, methamphetamine-induced memory deficits were associated with the decline in activity of GABAergic neurons caused by the decrease in the *α*2 subunit of GABA A receptors and GAD protein levels in the nucleus accumbens [169, 170].

Generally, alcohol causes a decreased inhibitory and an increased excitatory activity in neuronal networks leading to stronger dopamine output in the ventral striatum [171]. The decrease in GABAergic transmission level was observed in the striatum of alcoholic brain [172]. In alcohol-dependent rats, dopamine modulates GABA A receptors [173] and tonic current mediated by these receptors in medium spiny neurons of the nucleus accumbens [174]. In addition, the prolonged ethanol consumption revealed disinhibition in the striatum mediated by GABA A receptors, although synaptic transmission in the nucleus accumbens was unchanged [175].

Addictions are strongly associated with disruptions of the circadian clock. Drug and alcohol dependence are accompanied by changes in the daily rhythmicity [176–178] and the response to drugs or alcohol has different sensitivity over the light/dark cycle [179–181]. Cocaine can induce or restrain cyclic expression of some clock genes in the dorsal striatum [182, 183].

The disruption in circadian rhythmicity is also correlated with mood disorders [184, 185] which can also be induced by mutations of clock genes [186].

Since all disorders associated with the dopaminergic system in the striatum demonstrate a decrease in GABAergic transmission and reveal changes in the circadian rhythmicity, the involvement of inhibitory interneurons in striatum-related pathology seems to be highly probable.

## 9. Conclusions/Concluding Remarks

Despite the fact that GABAergic interneurons form diverse networks in different regions of the brain controlled by the circadian clock, they generally perform similar and, moreover, crucial role in all circuits. Inhibitory interneurons are involved in the generation of synchronized circadian oscillations and they are responsible for control of the excitation/inhibition balance. Unfortunately, information concerning diurnal or circadian changes of the particular types of inhibitory interneurons located in different brain structures is highly limited. Further studies of cyclic changes focused on individual interneuron classes could significantly contribute to the understanding of circadian clock mechanisms of their control.
